# Women Physicians in Academic Medicine of Japan

**DOI:** 10.31662/jmaj.2021-0116

**Published:** 2022-06-24

**Authors:** Sumiyo Akazawa, Yuki Fujimoto, Mio Sawada, Tsugiyasu Kanda, Takeshi Nakahashi

**Affiliations:** 1 Department of General Internal Medicine, Kanazawa Medical University, Uchinada, Japan; 2 Department of Respiratory Medicine, Kanazawa Medical University, Uchinada, Japan; 3 Department of Community Medicine, Kanazawa Medical University, Uchinada, Japan

**Keywords:** women physician, skill education, work sharing, childcare, work-life balance, sponsorship

## Abstract

Japan is a well-qualified country in promoting scientific advancement, but female scientists are too few in academic medicine positions. The government of Japan announced that the share of women in leadership positions accounted to at least 30% by 2020 in all fields in society. The number of female students also increased, but it was not much higher than other Organisation for Economic Co-operation and Development (OECD) countries. Women students always have higher passing frequencies in national examination for medical practitioners in Japan. The potential gap between physician gender and academic advancement is mentioned in any medical fields. Women physicians in academic medicine position are still few. For women physicians, medical and familial situations are inversely affected by the coronavirus disease 2019. We propose the recommendations to support women physicians’ right in academic medicine, accordingly to patients’ benefit. Women may be stepping up and leading efforts without titles or positions in ways that are significant and meaningful for their group or organization.

## Background

Let us remember that among Japanese women physician, Ine Kusumoto (1827-1903) was the first female physician who learned the western medical science, Ginko Ogino was the first licensed and practicing woman physician of western medicine, and Yayoi Yoshioka was a physician, educator, and women’s rights activist and founded the Tokyo Women’s Medical University in Japan. Their vitality and message could influence the recent gender problems in building a respectful equality in each member of academic medicine in Japan.

## Introduction

Gender gap in medical schools of Japan was revealed in 2018. Some medical universities have been systematically tampering its entrance exam scores to reduce the number of female students. The government investigated the medical faculties, and initial reports suggest that such gender discrimination is widespread in medical faculty admissions ^(1)^. Japan is a well-qualified country in promoting scientific advancement, but female scientists are too few in positions of authority who can help younger women with career enhancement. Finally, female scientists often avoid competition and underestimate their ability, leading to passivity when seeking leadership roles ^(2)^.

Progressive initiatives of empowering network for diversity started in 2018 as expansion of women’s participation in policy and decision-making processes in all fields in society of cabinet office in the government of Japan ^(3)^. Their aim is the share of women in leadership positions to at least 30% by 2020 in all fields in society, including politics, national and local civil services, private sector, education, and research.

The low number of women physicians in academic medicine is still one of the major problems in Japan compared with other countries. We investigated the gap or disparity from entrance examination to academic opportunities even with the COVID-19 pandemic. We reviewed several points of gender gap in medical school from entrance examination to faculty and finally in women physician even with the COVID-19 pandemic.

## 1. Gender Gap in Entrance Examination of Medical School

**Figure 1.**
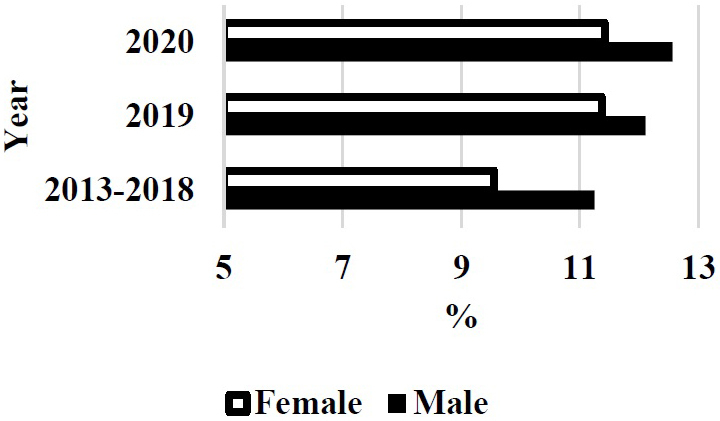
Gender difference of acceptance rate in the entrance examination of medical school of Japan. Female acceptance rate was increased in 2019 and 2020 compared with those in 2013-2018.

**Figure 2.**
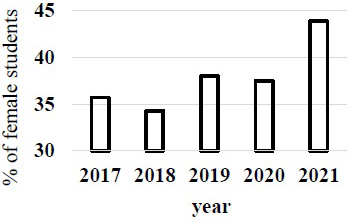
The frequency of female students in the first year of medical school of Japan. The rate was apparently increased in 2021 and became over 40%.

The Ministry of Education, Culture, Sports, Science and Technology (Japan) pointed out the gender gap in entrance examination of medical school in 2018. The rates of female students in 2019 and 2020 apparently increased after this trouble in Japan medical schools (Figure 1) ^(4)^. The prevalence of female students in the first year became higher in 2021 (Figure 2) ^(4)^.

**Figure 3.**
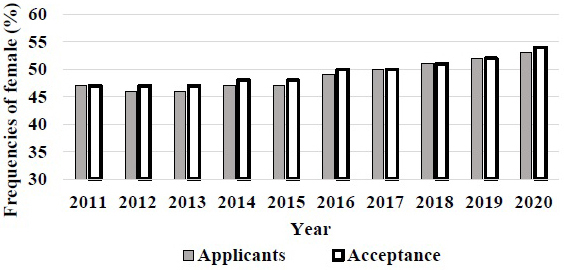
The frequencies of female entrance in US medical school. Both female application and entrance rate became over 50% since 2018.

Government investigation may affect the revision of gender gap in Japan medical schools after trouble in 2018. The number of female students also increased, but it was not much higher than other OECD countries ^(5)^. In the United States, the prevalence of women students accepted in medical school gradually increased since 2011 and reached over 50% in recent years (Figure 3). The gender gap in entrance examination of medical school will be changed in Japan. However, the problem may not only be the rate of gender gap but also the prejudice from society and medical educators for the coming generation ^(5)^.

## 2. Gender Gap in Medical Students and National Examination of Physician

**Figure 4.**
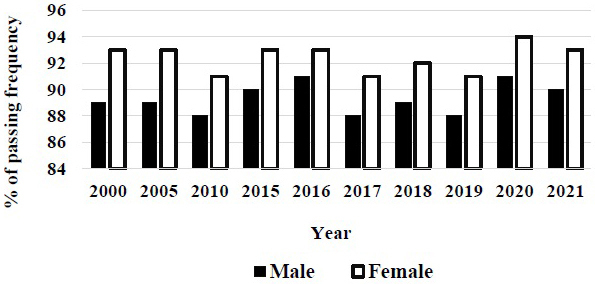
Gender difference in passing frequencies of the national examination for medical practitioner of Japan. Females always have higher frequency than male.

The gender gap is solving the acceptance of entrance examination in medical school of Japan, although female students are still lower number than male ones in medical school. Compared with men, women students always have higher passing frequencies in the national examination for medical practitioner in Japan (Figure 4) ^(4)^. The excellence of women students is recognized in passing the national examination in Japan. The composition of medical school classes reflects the gender balance in the US population, where 50.6% were women ^(6)^. The excellent points in women physician were reported. The microsurgical skills of male and female medical students are similar. Thus, regardless of gender, equal opportunities in the eventual pursuit of the surgical specialties should be available ^(7)^.

Even before residency, one student out of two is suffering from burnout ^(8)^. The high level of distress in the medical population from 24 studies. In a systematic meta-analysis, female students were more likely to be depressed than male. Depression affects almost one-third of medical students globally, but treatment rates are relatively low ^(8)^. In Germany, currently two out of three medical students are female. Medical students show a significantly higher prevalence of stress-related mental disorders than the population in general. Female students showed significantly higher values for depression as well as for emotional and cognitive burnout and significantly lower mental quality of life was ^(9)^.

## 3. Women Physician in Medical School

**Figure 5.**
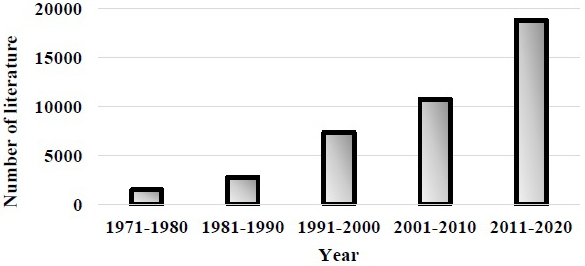
Number of publications with keywords “women physician” by PubMed. These were dramatically increased in these decades.

Publication of keywords “women physician” dramatically increased in recent decades (Figure 5).

**Figure 6.**
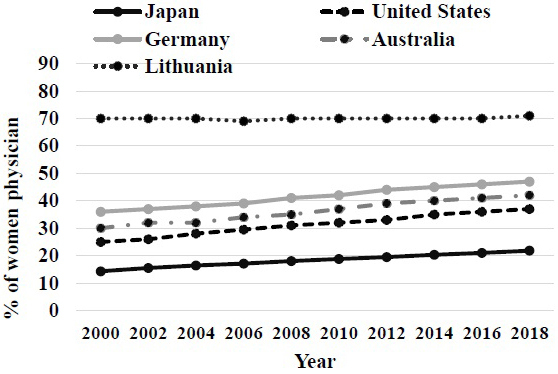
Frequent occupation of female physicians. The rate in Japan is still lower than that in other countries.

The occupation of female physicians of Japan is continuously lower compared with other countries (Figure 6) ^(10)^. The apprehension of the time-lag of the measure having affected the low number of physicians of our country was shown. A female physician is apt to have specific special specialties, such as in ophthalmology and a dermatology. In addition, the hurdle of reappointment after marriage is high for a female physician. Especially the percentage to part employment or clinic general practitioners that moves out has a female physician higher than a virile physician. The work-life balance support system of a medical doctor in hospitals is insufficient ^(11)^.

The potential gap between physician gender and academic advancement is mentioned in many medical fields ^(12), (13), (14), (15), (16), (17)^. Despite women composing half of current medical school classes, surgical specialties still struggle to attract and retain women. Opportunities for women in surgery have improved, although much work remains to make the surgical workplace supportive of women, empowering them to optimally contribute. These efforts will benefit organizations, the community, and future generations of surgeons and most importantly, profoundly, and positively affect the care of patients ^(18)^.

Articles in which both the first and last authors were women used at least 1 of the 25 positive terms in 10.9% of titles or abstracts versus 12.2% for articles involving a male first or last author, corresponding to a 12.3% relative difference ^(19)^. The paper found that (i) the female-authored papers are 1%-6% better written than equivalent papers by men, (ii) the gap is two times higher in published articles than in earlier draft versions of the same papers, and (iii) women’s writing gradually improves but men’s does not, which means that the readability gap grows over authors’ careers ^(20)^. There are no analysis of women physicians to contribute to journal authors compared with male physicians in Japan.

The prevalence of women physicians in academic medicine position increased from 23.9% in 2014 to 27% in 2019 in Japan ^(21)^. The ratio of professors in medicine also increased from 2.5% to 4.3%; however, the ratio is still low compared with other countries. Last year, 18% of US medical school deans and 19% of clinical department chairs were women, with only small increases in the past decade ^(22)^. That same year, fewer than 15% of academic departments of anesthesiology, emergency medicine, neurology, otolaryngology, and surgery were led by women, despite far greater proportions of senior women faculty ^(6)^. In 2018, one-third or fewer of senior associate deans, center directors, division chiefs, and section chiefs were women ^(11)^. Women increasingly served as assistant and associate deans in 2018 but were rarely appointed to oversee high-stakes and highly influential clinical or research missions in the analysis of 134 US medical school ^(22)^. This report showed that fewer women than expected were promoted to the rank of associate or full professor or appointed to the post of department chair, even after controlling for factors such as age, specialty, number of hours worked, and practice characteristics. Women comprised nearly half of residents and fellows in training, and most graduate students who enrolled in doctoral programs in the biological and medical sciences in the United States were women. Over the past decade, the proportions of women faculty in clinical and basic science academic departments have grown steadily. Nearly half of new faculty hires across all of academic medicine were women ^(6)^.

Recent data indicate that a smaller proportion of women attain senior rank in academic medicine compared with men, and men are more commonly promoted in 7 years in both clinical and basic sciences in the United States ^(23)^. Moreover, for many years, women have been less likely to successfully apply for National Institutes of Health (NIH) R01 grant renewals. Few editors of journals in education and in scientific disciplines are women. In addition, women are underrepresented as authors in the literature. A 2017 review^11^ of over 1.5 million medical research articles found that 35% of authors per author group were women. Approximately 40% of first authors and 27% of last authors were women. It is no wonder that the percentage of women who leave academic medicine has steadily increased each year, accounting to 41% in 2019 ^(24)^. A major report^13^ from 2018 indicates that half of women in medical school have experienced sexual harassment, most commonly gender-based harassment, and even more women faculty, cumulatively, have these experiences across the course of their careers in medicine ^(25)^.

Medical trainee burnout was reported in Japanese women physician. A cross-sectional study was showed 18 to 33% at a Japanese teaching hospital. In 2018, the percentages of participants with burnout in surveys were 33.3% ^(26)^. This report mentioned that medical educators might need to consider not only working hours but also individual job quality and satisfaction to address burnout. Only 6%-7% of Japanese people in the general population are depressed, which is substantially lower than the 25.2% reported among Japanese resident physicians. Taking these results together with the high burnout rates among Japanese resident physicians, we can expect a higher rate of overlap between burnout and depression in the participants as well ^(26)^.

Women physicians reported a wide variety of risk factors for mental and physical illness than med do and have poorer health outcome. This also applies to medical students as well ^(27)^. Women in academic medicine were more likely to report inadequate sleep, rare refreshment when waking, and excessive sleep in waking hours ^(17)^. A meta-analysis of physician suicide demonstrated a significant suicide risk for female physicians more than male physicians of the same age ^(27)^. The contributing factors analyzed as interventions specific to preventing physician burnout in women should include (1) addressing barriers to career satisfaction, work-life integration, and mental health; (2) identification and reduction of gender and maternal bias; (3) mentorship and sponsorship opportunities; and (4) family leave, lactation, and childcare policies and support. In addition, gaps in research must be addressed in an effort to inform best practices for measuring and pointing burnout among women physicians ^(28)^.

Physician mothers face unique challenges related to family planning, pregnancy, childcare, and work-life integration, which may have serious widespread implications. There is a paucity of available information on the extent and ramifications of such challenges and related solutions. Physician mothers face challenges that have negative implications for individuals, organizations, the healthcare system, and society. Clear understanding of associated challenges and potential solutions is a critical first step to address biases and barriers affecting physician mothers ^(29)^.

To enact sustainable change for gender equality, it is necessary to acknowledge and solve the complexity. Athena SWAN is the single most comprehensive and systemic gender equality scheme in Europe. It can be further strengthened by promoting the integration of sex and gender analysis in research and education. Gender equality policies in the wider European Research Area can benefit from exploring Athena SWAN’s 10 contextually embedded systemic approach to dynamic action planning and inclusive focus on all genders and categories of staff and students ^(30), (31)^.

The excellent intervention of women physician was reported. Elderly hospitalized patients treated by female internists have lower mortality and readmissions compared with those cared for by male internists. These findings suggest that the differences in practice patterns between male and female physicians as suggested in previous studies may have important clinical implications for patient outcomes ^(32)^. The higher mortality among female patients who are treated by male physicians. Female physicians have more success treating female patients ^(33)^. Considering the largest univariate effects, females scored higher in sensitivity, anxiety (apprehension), warmth, and complexity (openness to change), whereas males were higher in dutifulness (rule consciousness), emotional stability, and assertiveness (dominance) ^(34)^. These characteristic features may affect patient care, research aggressiveness, and literature writing.

## 4. Women Physician during the COVID-19 Pandemic

The coronavirus disease 2019 (COVID-19) pandemic has affected every facet of our work and personal lives. The COVID-19 pandemic may intensify workplace inequities for women and propose solutions for academic medicine ^(35)^. For women physicians, medical and familial situations are inversely affected by the COVID-19 pandemic. Close to half of all physicians are treating patients through telemedicine, up from 18% in 2018, while many plan to change jobs, opt out of patient care roles, or retire in response to the COVID-19 epidemic ^(36)^. New challenges triggered by the COVID-19 pandemic probably have exacerbated existing trends. Submissions of academic papers by female faculty members have decreased, while those of male faculty members have remained relatively unchanged ^(37)^.

During the COVID-19 pandemic, many governments have closed schools and implemented social distancing requirements that limit options for childcare while simultaneously requiring researchers to work from home. Robust evidence suggests that women in academic medicine shoulder more of the burden of domestic labor within their households than do men. Recent research also suggests that women in academia take on more domestic responsibilities than men, even in dual-career academic couples. Moreover, the productivity cost is higher for women. The qualitative investigation highlights the incidence of an “invisible burden” in self-identified dual parenting families ^(33)^.

Gender gap was reported in the published medical research literature. The results of an analysis were compared with a sample of 85,373 papers published in the same journals in 2019 ^(35)^. The COVID-19 pandemic might have amplified this gender gap in the medical literature. The restricted access to childcare and increased work-related service demands might have taken the greatest toll on early-career women, particularly early on when the disruptions were most unexpected, although the observational data cannot conclusively support causal claims ^(38)^. The gender gap, i.e., the percentage of articles on which men versus women were first authors, widened by 14 percentage points during the COVID-19 pandemic, despite many pertinent research fields showing near equal proportions of men and women first authors publishing in the same fields before the pandemic ^(39), (40)^.

Gender gap could produce a substantial cohort effect, especially for women with younger children, which is also the same for women physicians. The National Academies of Sciences, Engineering, and Medicine undertook a fast-track study on the effect of COVID-19 on the careers of women in science, technology, engineering, mathematics, and medicine ^(41)^.

## 5. Strategy for Women Physician in Academia

**Figure 7.**
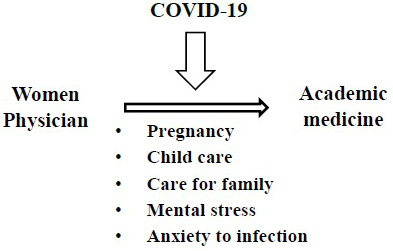
Schematic diagram of obstacles against the COVID-19 crisis for women physicians to academic medicine. Women-specific processes to academic medicine will be influenced by the COVID-19 crisis.

A 2021 report on gender equality in the EU shows the negative impact of the COVID-19 pandemic on women ^(42)^. The pandemic has exacerbated existing inequalities between men and women in almost all areas of life ^(42)^. Women’s inclusion in crisis response decision-making is crucial to address gender biases in decision-making and to ensure effective responses to the COVID-19 pandemic (Figure 7).

The consultation systems, such as educational programs ^(43), (44)^, mentorship ^(45)^, and sponsorship ^(46), (47)^, can support work-life balance or in-hospital childcare and promote hiring rate or promotion of women. Skills education is planned by new guidelines in surgical education ^(43)^, and artificial intelligence-based methodologies have the potential to improve clinical setting ^(47)^.

The reasons of gender gap include several barriers including “jock” culture, pregnancy concerns, a few mentors, and a few role models ^(48)^. The acknowledgment at individual and institutional levels can bring more women in the academic medical field. This will benefit not only women physicians but also patients as well.

**Figure 8.**
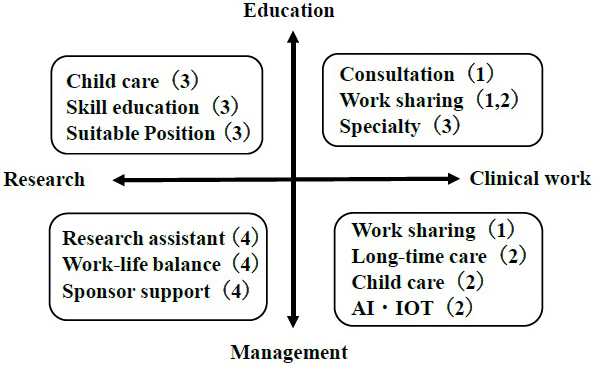
Schematic diagram of support suggestions for women physicians in academic medicine. The number in parenthesis shows the text of “5. Strategy for women physician in academia.”

We propose the following recommendations to support women physicians in academic medicine (Figure 8): (1) direction to education and clinical work, which includes specialty support, work sharing, and consultation system for female physician; (2) direction to clinical work and management, which includes work sharing, childcare resources, and new processes by artificial intelligence, Internet of Things, and long-time care for female physician; (3) direction to education and research, which includes skill management, childcare resources, and modified position for female physician; and (4) direction to management and research, which includes collaborative social networks, work-life balance, and sponsored support for female physician.

The more generalized support systems will be necessary for women physicians in academic medicine. First, we can analyze the mechanism for lack of academic proactivity. This period of decreased academic productivity may disproportionately derail progress toward promotion for women. Second, we must make efforts in the clinical or administrative contribution. Women may be stepping up and leading efforts without titles or positions in ways that are significant and meaningful for their group or organization. Many institutions struggled to support strong diversity, inclusion, and equity efforts prior to the COVID-19 pandemic ^(36)^.

## Conclusion

Although female scientists are too few in positions in academic medicine, we recommend definite support systems, such as educational systems, mentorships, and sponsorships, to make women physicians step forward in academic positions against prejudice of female-specific challenges. This thesis is not only for females but for handicapped members, such as those with physical, psychological, sensory, learning, or chronic health disabilities in academic medicine.

## Article Information

### Conflicts of Interest

None

### Author Contributions

SA, YF, and MS collected the references and structured this paper. TK reviewed this paper. TN managed and gave the various ideas for making this paper.
